# Supplementary material for: Neutrophil extracellular trap-associated RNA and LL37 enable self-amplifying inflammation in psoriasis
Source: Nat Commun. 2020 Jan 8;11:105. doi: 10.1038/s41467-019-13756-4 (PMC6949246; doi:10.1038/s41467-019-13756-4)
Supplement: Supplementary file 1 — Supplementary Information, Figures and Tables [file 41467_2019_13756_MOESM1_ESM.pdf]

SUPPLEMENTARY INFORMATION

Neutrophil-extracellular trap-associated RNA (naRNA) and LL37 enable self-amplifying inflammation in psoriasis

By Herster et al.

Supplementary Figures

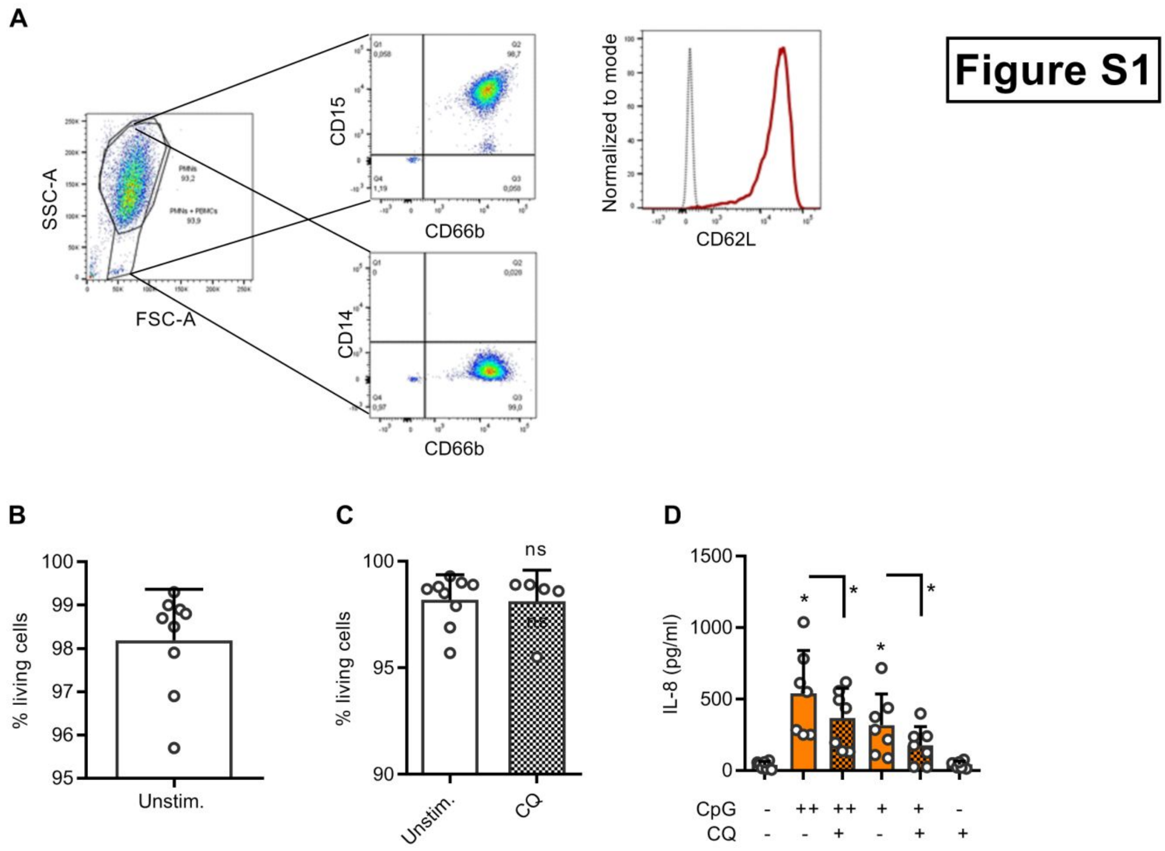

Supplementary Figure 1: Viability of primary PMNs is not compromised during *in vitro* treatments

(A) FACS analysis of from whole blood isolated PMNs. CD66b^{high}CD15^{high}CD14^{low} events were considered as PMNs. CD62L-positivity indicates cells were not pre-activated directly after isolation. Aqua Live-dead flow cytometric viability analysis of unstimulated PMNs after 4 h in culture (B, n=9 combined from several experiments) and including chloroquine (CQ) 30 min pre-incubation (C, n=5-8). (D) as in B but including CpG stimulation and ELISA analysis as indicated (n=7). In A one example representative of several donors is shown. B-D represent combined data (mean+SD) from ‘n’ biological replicates (each dot represents one donor). * p<0.05 according to Wilcoxon signed rank sum (C) and one-way ANOVA with Sidak correction (D). Source data is provided as a Source data file.

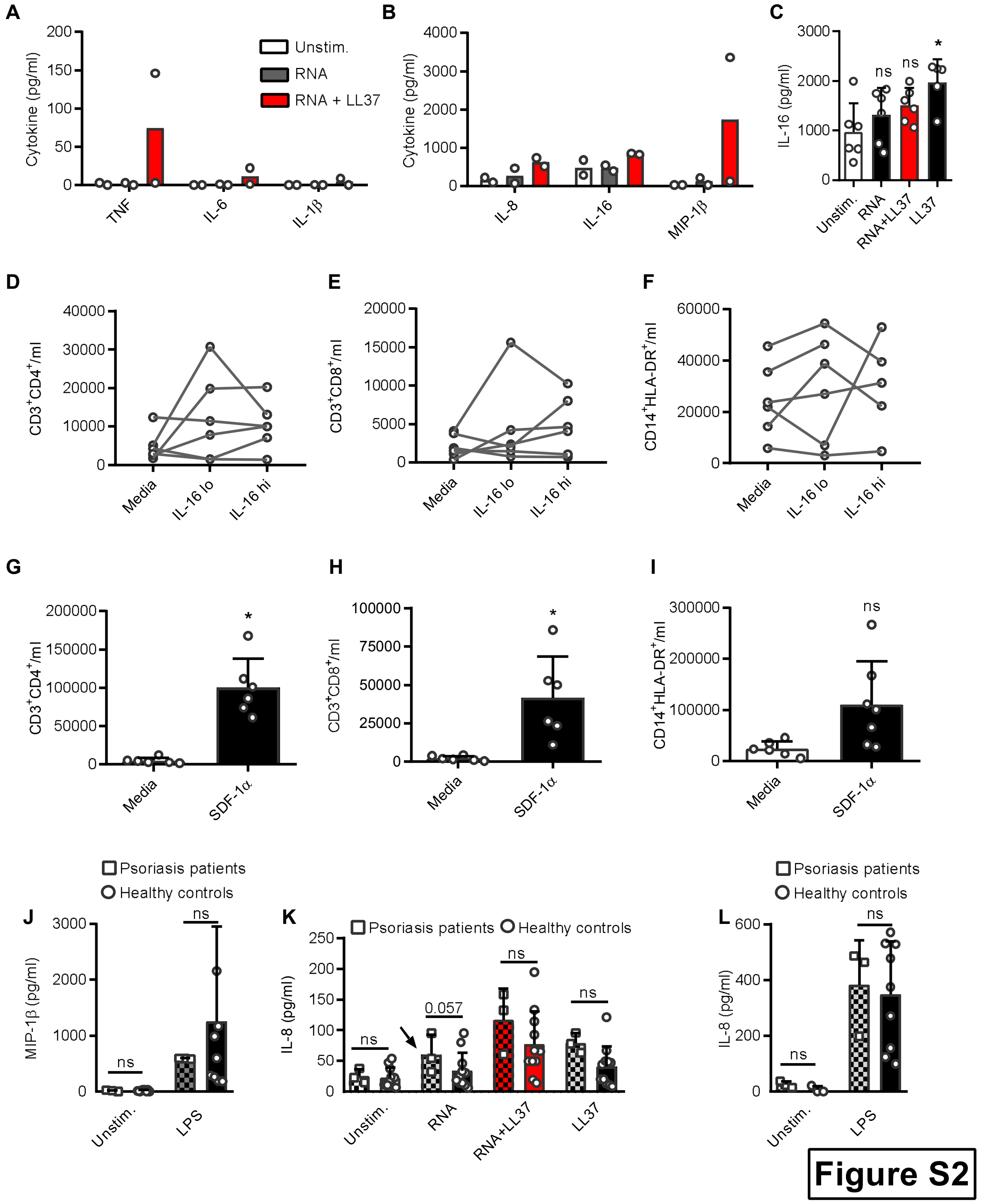

Supplementary Figure 2: Cytokine and migration responses in treated PMNs

(A, B) Luminex multiplex cytokine analysis of PMN supernatants (screening analysis). Mean values of TNF-α, IL-1β, IL-6, IL-16 and MIP-1β for n=2 donors shown. (C) IL-16 secreted from PMNs stimulated for 4 h as indicated (n=6). (D-I) Flow cytometric cell count of migrated $CD4^+$ T cells (D,G), $CD8^+$ T cells

(E,H) and $CD14^{+}HLA\text{-}DR^{+}$ monocytes (F,I) quantified in transwell assays with total PBMCs in the upper and (D-F) IL-16 (300 – 1500 pg/ml) or (G-I) SDF-1α (positive control, 100 ng/ml) in the lower compartment (n=6-7, p>0.05 for treatments vs. media). ELISA of MIP-1β (J) or IL-8 (K, L) secreted from psoriasis PMNs (n=3) or PMNs from sex-and age-matched healthy donors (n=3-9) in response to LPS (J, L) or RNA-LL37 complex (K) treatment. Only responses to treatment compared. Combined data (mean+SD) from 'n' biological replicates (each dot represents one donor) throughout. * p<0.05 according to Friedmann test with Dunn's correction (D, E, F), Student's t-test (G-I) or Kruskall-Wallis test with Dunn's correction (C, J-L). Source data is provided as a Source data file.

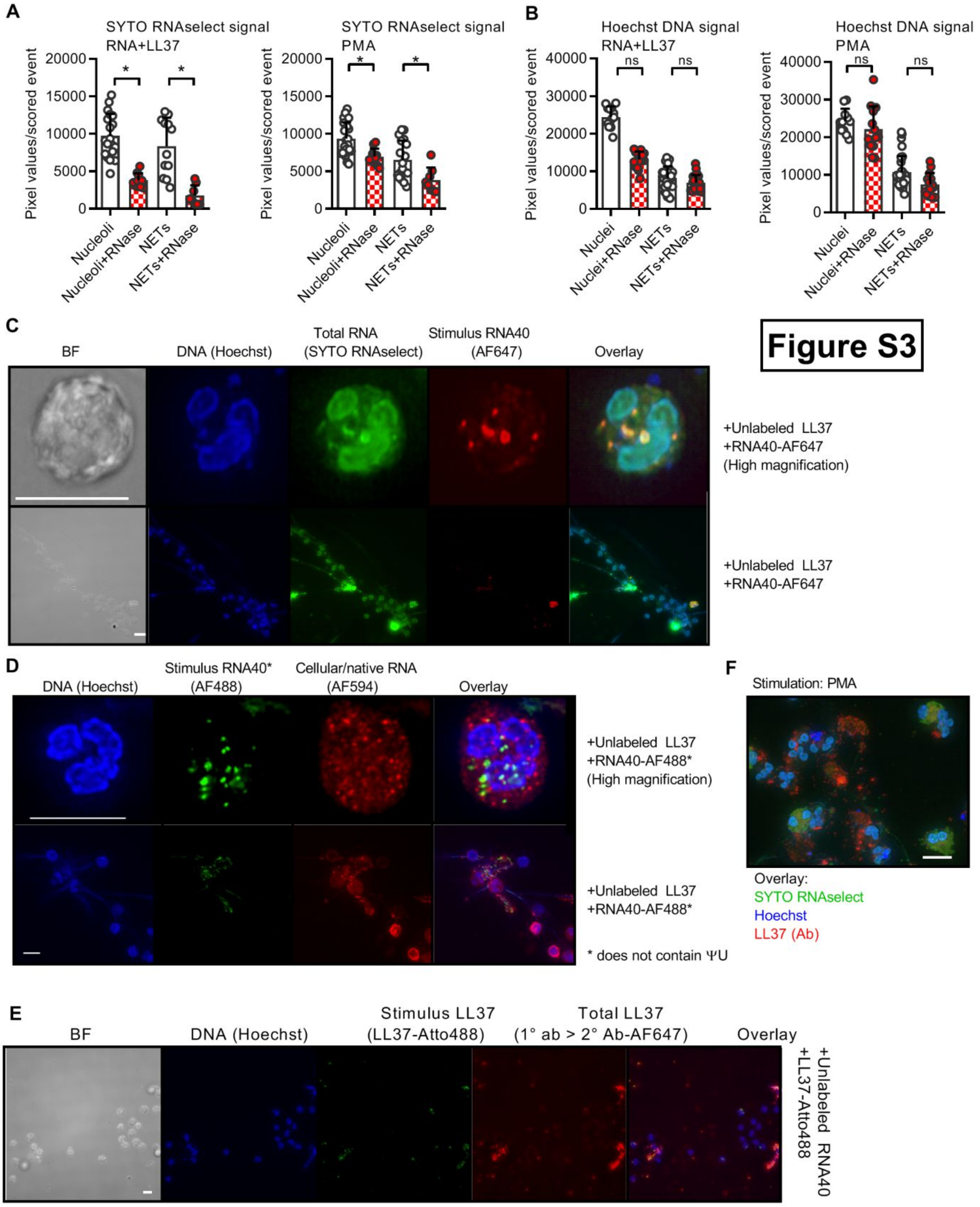

Supplementary Figure 3: RNA staining in PMNs and skin sections is specific

(A, B) Specificity of SYTO RNAselect staining: Fixed NETs were treated with RNase A or buffer control (n=4) and quantification of pixel values of nuclear or NET events scored by a blinded observer in the RNA (A) or DNA channels (B) is shown for one representative experiment. Microscopy analysis of PMNs stimulated with either RNA-Alexa647 (C) or RNA-Alexa488 (D) and unlabeled LL37 (C, D) or unlabeled RNA and LL37-Atto488 (E) to distinguish exogenously added RNA (red in C, green in D) or

LL37 (green in E) from endogenously released RNA (green in C, SYTO RNAselect; red in D, anti-ΨU and Alexa594-conjugated secondary Ab) or LL37 (red in E, anti-LL37 and Alexa647-conjugated secondary Ab). n=2 for all experiments. (F) Staining for RNA in granule-like patterns observed for selected PMNs. In A and B combined data (mean+SD) from n biological replicates (each dot represent pixel values from one picture) are shown. In C-F one representative of 'n' replicates is shown. * $p<0.05$ according to one-way ANOVA with Holm-Sidak's correction (A) or Kruskall-Wallis test with Dunn's correction (B) to adjust for multiple testing. Source data is provided as a Source data file.

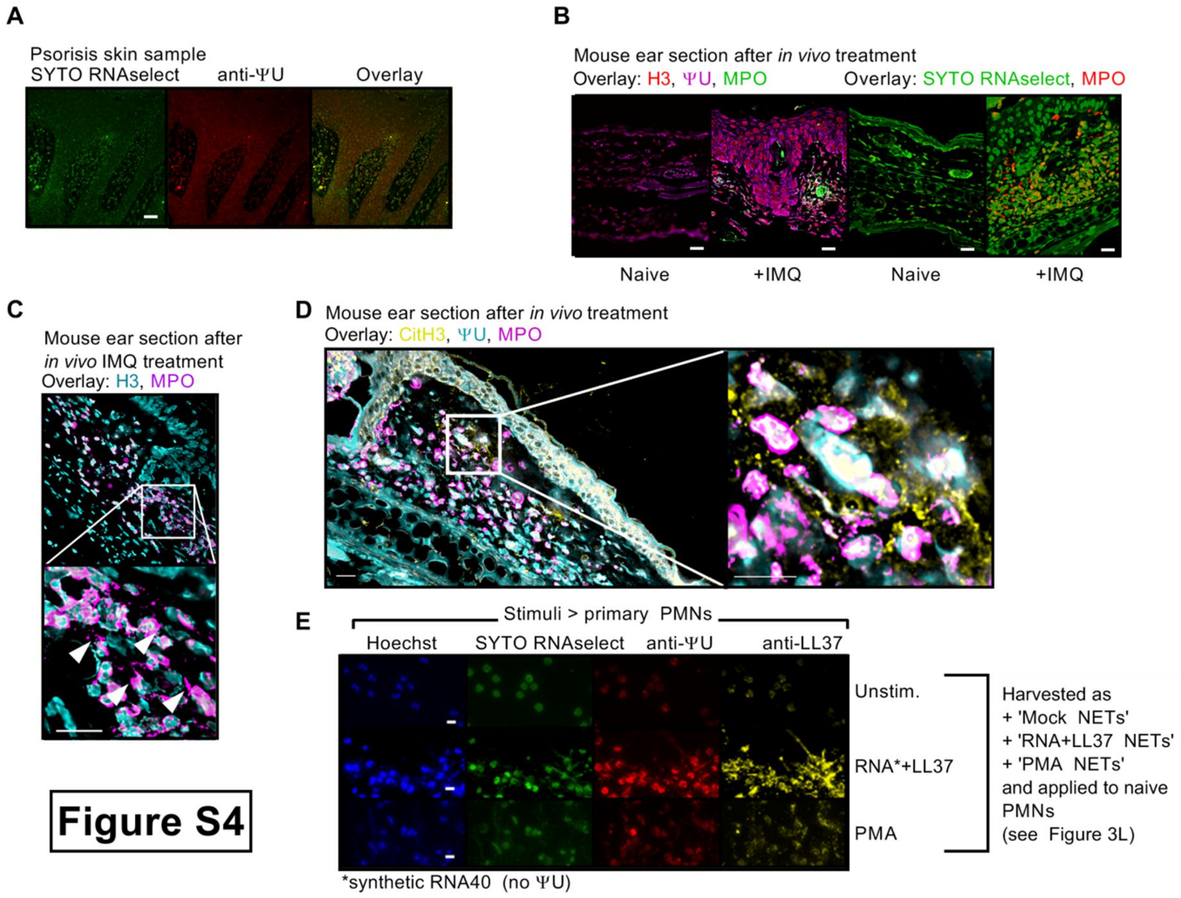

Supplementary Figure 4: RNA-LL37 in human psoriasis lesions and mouse ears *in vivo*

(A) SYTO RNAselect and anti-ΨU staining strongly overlap in human skin samples (n=12 patients and 3 healthy controls, scale bar = 20 µm). (B, C) IMQ-induced experimental psoriasis model: representative IF staining of ear sections from C57BL/6mice treated *in vivo* and subsequently stained as indicated (n=4-5 for each group, scale bar = 20 µm, close-up shown in C, arrowheads indicate NET-like structures). (D) Intradermal injections of mouse ears with RNA-LL37 complexes. Close-up shows extracellular RNA and citH3 (NET-like structures, scale bar = 20 µm, n=6). (E) Immunofluorescence of primary PMNs isolated from human healthy donors which released NETs (n=3; scale bar = 10 µm). These were harvested as 'Mock NETs', 'RNA+LL37 NETs' or 'PMA NETs', respectively, and applied to naïve PMNs (see Fig. 3L). In A-E one representative of ‘n’ replicates is shown.

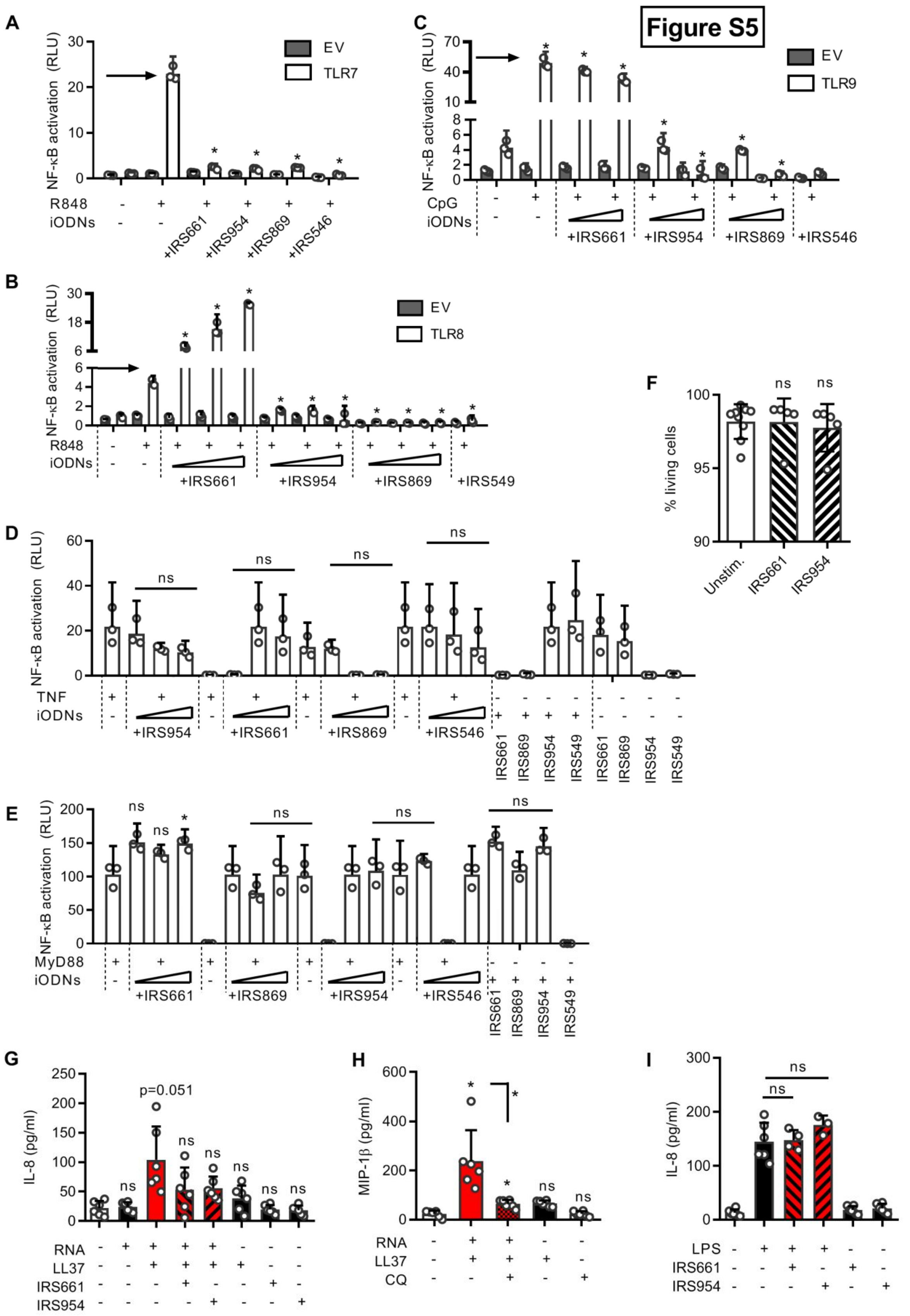
Figure S5
A
NF-κB activation (RLU)
EV
TLR7
R848
iODNs
+IRS661
+IRS954
+IRS869
+IRS546
B
EV
TLR8
+IRS549
C
EV
TLR9
CpG
D
TNF
E
MyD88
F
% living cells
Unstim.
IRS661
IRS954
G
IL-8 (pg/ml)
p=0.051
RNA
LL37
H
MIP-1β (pg/ml)
CQ
I
LPS

Supplementary Figure 5: Specificity of iODNs on TLR8-dependent signaling

(A-E) NF-κB dual luciferase reporter assay in HEK293 cells, transfected with NF-κB firefly luciferase reporter, *Renilla* control reporter and plasmids for either TLR7 (A), TLR8 (B), TLR9 (C) or MyD88 (E) or an equivalent amount of empty plasmid (EV) (D), subsequently stimulated with R848 (A, B), CpG ODN (C), recombinant TNF (D) or left unstimulated (E), without (arrow) or with IRS661, IRS954, IRS869 and IRS546 (n=2 each). Arrows indicate the level of stimulation observed in the absence of IRS. (F) Aqua Live-dead flow cytometric viability analysis of PMNs treated with IRS661 and IRS954 only (4 h, n=5-9). (G, I) IL-8 release from PMNs stimulated with RNA-LL37 complexes (G, n=6) or LPS (I, n=4-6) with or without IRS661 (1 nM), IRS954 (50 nM) pre-incubation (30 min) quantified by ELISA. (H) as in G but stimulation with RNA-LL37 complexes and pre-incubation (30 min) with chloroquine (CQ, 10 µM) and measuring MIP-1β release (n=6). F-H represent combined data (mean+SD) from 'n' biological replicates (each dot represents one donor). In A-E one representative of 'n' replicates is shown (mean+SD). * $p<0.05$ according to two-way ANOVA with Dunnett's correction (A-C), one-way ANOVA with Dunnett's correction (only TNF or MyD88 group, respectively, in D, E), Kruskall-Wallis test with Dunn's correction (F) or one-way ANOVA with Sidak correction (G, H, I). Source data is provided as a Source data file.

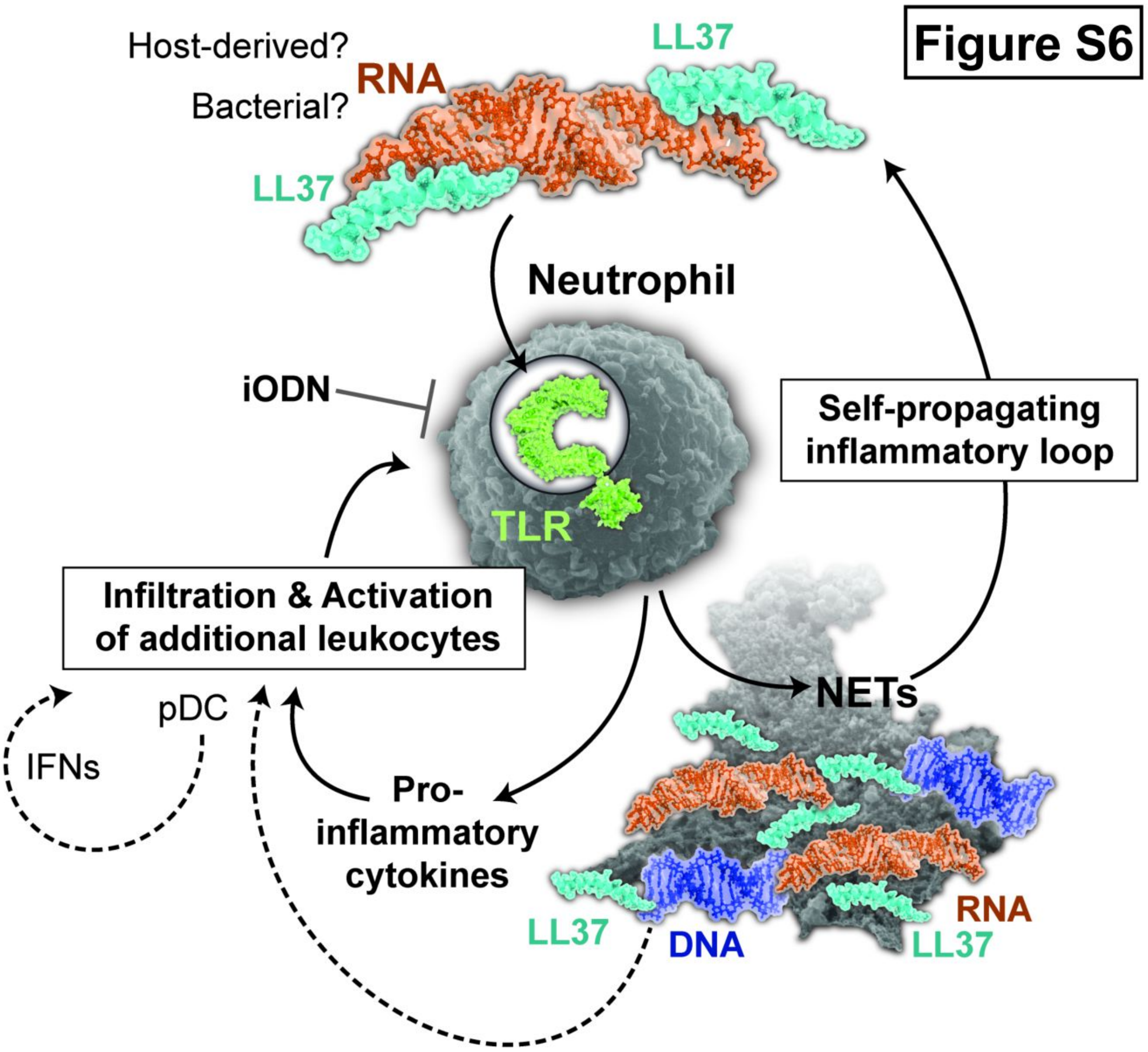

Supplementary Figure 6: Graphical summary of key findings

By releasing cytokines and RNA-LL37-containing NETs PMNs may fuel a self-propagating inflammatory cycle in psoriasis.

Supplementary Tables

Supplementary Table 1: Summary of primary PMN responses across experiments.

Analyte	LPS	PMA	R848	RNA	Pam2	Pam3	CpG DNA	genomic DNA/ssDNA	RNA + LL37	genomic DNA/ssDNA + LL37
IL-8	++	+++	++	+/-	+++	++	+++	-	++	-
CD62L	+++	+++	-	-	+++	++	+++	-	+	-
ROS	+/-	+++	-	-	-	-	-	-	-	-

Supplementary Table 2: Commercial TLR ligands and inhibitors

Component	company	Product
LL37	InvivoGen	tlrl-l37
LPS-EK (ultrapure)	InvivoGen	tlrl-peklps
R848 (Resiquimod)	InvivoGen	tlrl-r848-5
Chloroquine	InvivoGen	tlrl-chq
PMA	InvivoGen	tlrl-pma
Pam2CSK4	InvivoGen	tlrl-pm2s-1
Pam3CSK4	InvivoGen	tlrl-pms

Supplementary Table 3: RNA/DNA and inhibitors

Component	Sequence	company
CpG2006	5'TsCsGsTsCsGsTsTsTsTsGsTsCsGsTsTsTsTsGsTsCsGsTsT3'	TIB
RNA40	5'GsCsCsCsGsUsCsUsGsUsUsGsUsGsUsGsAsCsUsC3'	iba
ssDNA60	5'AC(AC)$_{28}$AC3'	TIB
IRS546	5'TsCsCsTsGsCsAsGsGsTsTsAsAsGsT3'	TIB
IRS661	5'TsGsCsTsTsGsCsAsAsGsCsTsTsGsCsAsAsGsCsA3'	TIB
IRS869	5'TsCsCsTsGsGsAsGsGsGsTsTsGsT3'	TIB
IRS954	5'TsGsCsTsCsCsTsGsGsAsGsGsGsTsTsGsT3'	TIB

ssRNA40 was obtained from IBA. The backbone is phosphorothioate.

Supplementary Table 4: Antibodies and recombinant proteins

Item	fluorophore	species	isotype	company	Product no.
**Isotype control**	PE	mouse	IgG1 kappa	eBioscience	12471442
**Isotype control**	FITC	mouse	IgM	BioLegend	401605
**Isotype control**	APC	mouse	IgG1 kappa	BD Bioscience	550854
**Isotype control**	BV421	mouse	IgG1 kappa	BioLegend	400157
**Isotype control**	AF488	mouse	IgG2a kappa	BioLegend	400233
**Isotype control**	FITC	mouse	IgG2b	BioLegend	401206
**Anti-hCD15**	PE	mouse	IgG1 kappa	BioLegend	323006
**Anti-hCD66b**	FITC	mouse	IgG1 kappa	BioLegend	305103
**Anti-hCD62L**	BV421	mouse	IgG1 kappa	BioLegend	30482
**Anti-hCD14**	PE	mouse	IgG1 kappa	ImmunoTools	21620144
**Anti-hCD3**	AF488	mouse	IgG2a	BioLegend	317310
**Anti-hCD4**	PE	mouse	IgG1 kappa	BioLegend	300508
**Anti-hCD11b**	APC	mouse	IgG1 kappa	BioLegend	301310
**Anti-hCD19**	BV421	mouse	IgG1 kappa	BioLegend	302234
**Anti-hCD8**	APC	mouse	IgG1 kappa	ImmunoTools	21810086
**Anti-hHLA-DR**	FITC	mouse	IgG2b kappa	BioLegend	327006
**Recombinant hMIP-1β**	-	-	-	ImmunoTools	11343223
**Recombinant hIL-16**	-	-	-	ImmunoTools	11340163
**Recombinant hSDF-1α**	-	-	-	ImmunoTools	11343363
**SYTO RNAselect**	n/a	-	-	ThermoFisher	S32703
**Anti-hLL37**	unconjugated	rabbit	IgG	LSBio	LS-B6696-500
**Anti-ψU**	unconjugated	mouse	IgG1	MBL	MBL-D347-3
**Anti-hNeutrophil Elastase**	unconjugated	mouse	IgG1	Novus Biologicals	MAB91671-100
**Anti-h/m MPO**	unconjugated	goat	IgG	R&D systems	AF3667
**Anti-Histone H3**	unconjugated	rabbit	IgG	Novus Biologicals	NB500-171
**Anti-citrullinated Histone H3**	unconjugated	rabbit	IgG	abcam	ab5103
**Anti-rabbit IgG**	AF647	chicken	IgY	ThermoFisher	A-21443
**Anti-mouse IgG**	AF594	chicken	IgY	ThermoFisher	A-21201
**Anti-mouse IgG**	AF488	chicken	IgY	ThermoFIsher	A-21200
**Anti-goat IgG**	AF594	chicken	IgY	ThermoFIsher	A- 21468

Supplementary Table 5: Plamids used for HEK293T transfection

Plasmid name Insert	Vector backbone	Insert
EGFP	pC1-EGFP	EGFP
NF-κB reporter	pGL3	6x NFKB response element
Renilla	pRL-TK	Renilla
hTLR7	pcDNA3.1 (+)	hTLR7
hTLR8	pcDNA3.1 (+)	hTLR8
hTLR9	pEF-SEM	hTLR9
MyD88	pTO-N-SH Streptag N-terminal Gateway	hMyD88 FL aa13-296 L265P, Stop-codon
pcDNA3.1	pcDNA3.1 (+)	Empty
pEF-SEM	pEF-SEM	Empty

Supplementary Table 6: 10x Ammonium chloride erythrocyte lysis buffer

Compound	company	Product no.
1.54 M NH_4Cl	Roth	5470.1
100 mM $KHCO_3$	Fluka	60220
1 mM EDTA; pH 8	ThermoFisher	15575020
dissolved in Ampuwa water	Fresenius Kabi	1833
pH adjusted to 7.3, sterile filtered (0.22 µm)		
