# Supplementary material for: Neutrophil extracellular trap-associated RNA and LL37 enable self-amplifying inflammation in psoriasis
Source: Nat Commun. 2020 Jan 8;11:105. doi: 10.1038/s41467-019-13756-4 (PMC6949246; doi:10.1038/s41467-019-13756-4)
Supplement: Supplementary file 3 — Description of additional supplementary files [file 41467_2019_13756_MOESM3_ESM.docx]

Description of Additional supplementary files

File Name: Supplementary Movie 1

description: Live cell imaging of unstimulated PMNs isolated from a healthy donor. The cells were stained with Hoechst 33342 (nuclei, blue) and SYTO RNAselect (RNA, green). The cells were tracked for a total of 3 hours. The movie shows one representative donor of n=6.

File Name: Supplementary Movie 2

Description: Live cell imaging of RNA-LL37 complexes stimulated PMNs isolated from a healthy donor. The cells were stained with Hoechst 33342 (nuclei, blue) and SYTO RNAselect (RNA, green). The cells were tracked for a total of 3 hours. The movie shows one representative donor of n=6.

File Name: Supplementary Movie 3

Description: Live cell imaging of PMA (600 nM) stimulated PMNs isolated from a healthy donor. The cells were stained with Hoechst 33342 (nuclei, blue) and SYTO RNAselect (RNA, green). The cells were tracked for a total of 3 hours. The movie shows one representative donor of n=6.

File Name: Supplementary Movie 4

Description: 3D reconstruction of a psoriatic skin sample previously stained for immunofluorescence using an anti-LL37 antibody with subsequent secondary antibody (red) and RNAselect (green). The overlay of both signals shows a yellow signal. This movie shows one representative patient sample of n=12.
