# Supplementary material for: Neutrophil extracellular trap-associated RNA and LL37 enable self-amplifying inflammation in psoriasis
Source: Nat Commun. 2020 Jan 8;11:105. doi: 10.1038/s41467-019-13756-4 (PMC6949246; doi:10.1038/s41467-019-13756-4)
Supplement: Supplementary file 8 — Reporting Summary [file 41467_2019_13756_MOESM8_ESM.pdf]

nature research

Corresponding author(s): Alexander N. R. Weber

Last updated by author(s): Oct 30, 2019

Reporting Summary

Nature Research wishes to improve the reproducibility of the work that we publish. This form provides structure for consistency and transparency in reporting. For further information on Nature Research policies, see Authors & Referees and the Editorial Policy Checklist.

Statistics

For all statistical analyses, confirm that the following items are present in the figure legend, table legend, main text, or Methods section.

n/a	Confirmed	
☐	☒	The exact sample size (*n*) for each experimental group/condition, given as a discrete number and unit of measurement
☐	☒	A statement on whether measurements were taken from distinct samples or whether the same sample was measured repeatedly
☐	☒	The statistical test(s) used AND whether they are one- or two-sided<br>*Only common tests should be described solely by name; describe more complex techniques in the Methods section.*
☒	☐	A description of all covariates tested
☐	☒	A description of any assumptions or corrections, such as tests of normality and adjustment for multiple comparisons
☐	☒	A full description of the statistical parameters including central tendency (e.g. means) or other basic estimates (e.g. regression coefficient) AND variation (e.g. standard deviation) or associated estimates of uncertainty (e.g. confidence intervals)
☐	☒	For null hypothesis testing, the test statistic (e.g. F, t, r) with confidence intervals, effect sizes, degrees of freedom and P value noted<br>*Give P values as exact values whenever suitable.*
☒	☐	For Bayesian analysis, information on the choice of priors and Markov chain Monte Carlo settings
☒	☐	For hierarchical and complex designs, identification of the appropriate level for tests and full reporting of outcomes
☒	☐	Estimates of effect sizes (e.g. Cohen's d, Pearson's r), indicating how they were calculated

Our web collection on statistics for biologists contains articles on many of the points above.

Software and code

Policy information about availability of computer code

Data collection	FACS/CBA data were aquired using a BD FACS Canto II and the corresponding FACS Diva software. Imagestream data were aquired using an ImageStream X MKII with the INSPIRE instrument controller software (Merck/Amnis). For Luminex analysis a Luminex 100/200 instrument was used. In vivo bioluminescence data were acquired using an IVIS Lumina LT Series III imager. For microscopy a Nikon Ti2 eclipse and the Nikon software NIS Elements II was used. ELISAs were measured on a Molecular Devices Spectra Max Plus ELISA reader. Dual luciferase assays were measured on a Fluostar Optima instrument (BMG Labtech).
Data analysis	GraphPad Prism 7 and 8 (GraphPad Software, Inc.) was used to prepare graphs, to collect data and to perfrom statistical analysis on any numerical data from the following sources: FACS/CBA data were analyzed in and exported from FlowJo v10 and FCAP Array V3 (BD Bioscience). Imagestream analysis were performed using IDEAS V4 (Merck/Amnis) and microscopy analysis were performed in and exported from the Nikon software NIS Elements II and Fiji/ImageJ. Bioluminescence data were analyzed in and exported from Living Image software version 4.5. software. ELISA data were analyzed in and exported from Molecular Devices Softmax Pro 2.1.0 software. Dual luciferase data were analyzed in and exported from BMG Labtech MARS Data Analysis Software Version 1.2.

For manuscripts utilizing custom algorithms or software that are central to the research but not yet described in published literature, software must be made available to editors/reviewers. We strongly encourage code deposition in a community repository (e.g. GitHub). See the Nature Research guidelines for submitting code & software for further information.

Data

Policy information about availability of data

All manuscripts must include a data availability statement. This statement should provide the following information, where applicable:

- Accession codes, unique identifiers, or web links for publicly available datasets
- A list of figures that have associated raw data
- A description of any restrictions on data availability

The authors declare that all data supporting the findings of this study are available within the paper, its supplementary information files and/or the source data file

Field-specific reporting

Please select the one below that is the best fit for your research. If you are not sure, read the appropriate sections before making your selection.

☒ Life sciences ☐ Behavioural & social sciences ☐ Ecological, evolutionary & environmental sciences

For a reference copy of the document with all sections, see nature.com/documents/nr-reporting-summary-flat.pdf

Life sciences study design

All studies must disclose on these points even when the disclosure is negative.

Sample size	Depending on availability at least 4-6 donors or mice per group and experiment were used
Data exclusions	In case of extreme values, outliers were statistically identified using the ROUT method at high (0.5%) stringency; data were only include if severe and documented technical difficulties were experienced during sample acquisition or analysis
Replication	Experiments were replicated several times using several donors or mice and identical protocols and settings as indicated in each figure legend
Randomization	No formal randomization procedure was used. Animals were manually assigned to the experimental groups at random. Healthy blood donors were sex-and age matched and randomly assigned within these ramifications
Blinding	Most of the experimental analyses were performed without blinding but scoring of microscopy was carried out by a blinded independent observer

Reporting for specific materials, systems and methods

We require information from authors about some types of materials, experimental systems and methods used in many studies. Here, indicate whether each material, system or method listed is relevant to your study. If you are not sure if a list item applies to your research, read the appropriate section before selecting a response.

Materials & experimental systems

n/a	Involved in the study
☐	☒ Antibodies
☐	☒ Eukaryotic cell lines
☒	☐ Palaeontology
☐	☒ Animals and other organisms
☐	☒ Human research participants
☒	☐ Clinical data

Methods

n/a	Involved in the study
☒	☐ ChIP-seq
☐	☒ Flow cytometry
☒	☐ MRI-based neuroimaging

Antibodies

Antibodies used	Information given in Table S4
Validation	All antibodies were from commercial sources and were either validated by other groups or by us. They were additionally titrated by us or the recommended dilution was used from the corresponding data sheets.

Eukaryotic cell lines

Policy information about cell lines

Cell line source(s)	Academic laboratories
Authentication	None
Mycoplasma contamination	All cell lines used were tested prior to use and on a regular basis and showed no mycoplasma contamination
Commonly misidentified lines (See ICLAC register)	none

Animals and other organisms

Policy information about studies involving animals; ARRIVE guidelines recommended for reporting animal research

Laboratory animals	WT C57BL/6J mice between 8 and 16 weeks of age were used. For in vivo intradermal injections only male mice were used (between 8 and 12 weeks of age). Unc93b1 ko mice between 8 and 16 weeks of age were used. TLR13 ko mice between 8 and 16 weeks of age were used. LysM-eGFP mice 8 to 12 weeks of age were used. These animals were handled according to local official policies, procedures and animal experiment approvals as described in methods.
Wild animals	none
Field-collected samples	none
Ethics oversight	All mouse colonies were maintained in line with local regulatory guidelines and hygiene monitoring

Note that full information on the approval of the study protocol must also be provided in the manuscript.

Human research participants

Policy information about studies involving human research participants

Population characteristics	Healthy blood and skin donors and from psoriasis patients were obtained. Patients had a PASI score of over 10 and no systemic therapy at the moment of blood or skin sampling. To compare with patient's blood samples, sex-and age matched healthy controls were used. Skin sections were from 12 patients with Plaque Psoriasis and 1 patient with Psoriasis guttata.
Recruitment	Patients were recruited at the Department of Dermatology in Tübingen oder Heidelberg<br>Healthy donors were recruited at the Department of Immunology in Tübingen
Ethics oversight	All patients and healthy blood donors included in this study provided their written informed consent before study participation. Approval for use of their biomaterials was obtained by the local ethics committees at the University Hospitals of Tübingen and Heidelberg, in accordance with the principles laid down in the Declaration of Helsinki as well as applicable laws and regulations.

Note that full information on the approval of the study protocol must also be provided in the manuscript.

Flow Cytometry

Plots

Confirm that:

☒ The axis labels state the marker and fluorochrome used (e.g. CD4-FITC).

☒ The axis scales are clearly visible. Include numbers along axes only for bottom left plot of group (a 'group' is an analysis of identical markers).

☒ All plots are contour plots with outliers or pseudocolor plots.

☒ A numerical value for number of cells or percentage (with statistics) is provided.

Methodology

Sample preparation	After PMN isolation and stimulation, the purity and activation status of neutrophils was determined by flow cytometry. 200 µl of the cell suspension was transferred into a 96 well plate (U-shape) and spun down for 5 min at 448 x g, 4 °C. FcR block was performed using pooled human serum diluted 1:10 in FACS buffer (PBS, 1 mM EDTA, 2% FBS heat inactivated) for 15 min at 4 °C. After washing, the samples were stained for approximately 20-30 min at 4°C in the dark. Thereafter, fixation buffer (4% PFA in PBS) was added to the cell pellets for 10 min at RT in the dark. After an additional washing step, the cell pellets were resuspended in 150 µl FACS buffer.
Instrument	Measurements were performed on a FACS Canto II from BD Bioscience
Software	BD Bioscience, Diva software. Analysis was performed using FlowJo V10 analysis software.
Cell population abundance	more than 95% after isolation for PMNs.
Gating strategy	PMNs/PBMCs were gated by FSC-A/SSC-A and doublets were excluded. Live cells were gated using the following markers: in CD15/CD66b double positive cells and CD14 low/negative were considered to be human PMNs (CD62L was used as an early activation marker). For transwell experiments the PBMC gate was used. CD3/CD4 or CD3/CD8 double positive cells were considered to be T cells. CD14/HLA-DR double positive cells were considered to be monocytes.

☒ Tick this box to confirm that a figure exemplifying the gating strategy is provided in the Supplementary Information.
